# Thermal Limit for Metazoan Life in Question: *In Vivo* Heat Tolerance of the Pompeii Worm

**DOI:** 10.1371/journal.pone.0064074

**Published:** 2013-05-29

**Authors:** Juliette Ravaux, Gérard Hamel, Magali Zbinden, Aurélie A. Tasiemski, Isabelle Boutet, Nelly Léger, Arnaud Tanguy, Didier Jollivet, Bruce Shillito

**Affiliations:** 1 Adaptations aux Milieux Extrêmes, UMR CNRS 7138, Université Pierre et Marie Curie - Paris 06, Paris, France; 2 Institut de Minéralogie et de Physique des Milieux Condensés, UMR CNRS 7590, Paris, France; 3 Ecoimmunology of Marine Annelids, UMR CNRS 8198, Université de Lille 1, Villeneuve d’Ascq, France; 4 Génétique de l’Adaptation en Milieux Extrêmes, UMR CNRS 7144, Station Biologique de Roscoff, Université Pierre et Marie Curie - Paris 06, Roscoff, France; California State University Fullerton, United States of America

## Abstract

The thermal limit for metazoan life, expected to be around 50°C, has been debated since the discovery of the Pompeii worm *Alvinella pompejana*, which colonizes black smoker chimney walls at deep-sea vents. While indirect evidence predicts body temperatures lower than 50°C, repeated *in situ* temperature measurements depict an animal thriving at temperatures of 60°C and more. This controversy was to remain as long as this species escaped *in vivo* investigations, due to irremediable mortalities upon non-isobaric sampling. Here we report from the first heat-exposure experiments with live *A. pompejana*, following isobaric sampling and subsequent transfer in a laboratory pressurized aquarium. A prolonged (2 hours) exposure in the 50–55°C range was lethal, inducing severe tissue damages, cell mortalities and triggering a heat stress response, therefore showing that Alvinella’s upper thermal limit clearly is below 55°C. A comparison with *hsp70* stress gene expressions of individuals analysed directly after sampling *in situ* confirms that *Alvinella pompejana* does not experience long-term exposures to temperature above 50°C in its natural environment. The thermal optimum is nevertheless beyond 42°C, which confirms that the Pompeii worm ranks among the most thermotolerant metazoans.

## Introduction

Deep-sea hydrothermal vents are believed to host the most thermophilic microorganisms, and the actual upper thermal limit (UTL) for life was indeed recorded in hydrothermal Archae, which grow at temperatures up to 122°C [1]. Some vent animals also thrive close to the hydrothermal fluids and live at the edge of the UTL for metazoan life (50°C, [2]), like some of the alvinellid polychaetes, chimney dwellers found exclusively in association to high temperature venting. Discrete measurements have reported temperatures around 100°C [3], [4] in the close surrounding of the emblematic species *Alvinella pompejana* Desbruyères and Laubier 1980 [5]. Furthermore, continuous recordings inside the worm tubes witnessed of sustained temperature of 60°C, well beyond the metazoan UTL, with regular spikes above 80°C [6], [7]. Several studies on *A. pompejana* thence focused on both the thermostability and the optimal efficiency of its macromolecules, and while proving molecular performance similar or greater than for homeotherms, nevertheless suggested body temperatures below 50°C [8]. However, recent *in vitro* studies continuously present *in situ* thermal limit inference above 50°C as a start for molecular investigations while simultaneously emphasizing this *in vitro*/*in situ* discrepancy [9], [10], [11], [12], [13], [14]. Only the *in vivo* approach can solve this contentious issue, but so far this species hardly survives recovery from 2,500 meters depth, precluding the empirical determination of thermal limit on live specimens [8], [15], [16], [17], [18]. To minimize the collection and depressurization trauma, we developed a system of isobaric sampling and transfer of *A. pompejana* colonies towards a receiving high-pressure aquarium named BALIST, according to the project’s acronym (Biology of ALvinella, Isobaric Sampling and Transfer). This system allowed *in vivo* experimentation to be carried out under controlled temperature, at *in situ* pressure of 25 MPa, which subsequently provided the first empirical demonstration of *A. pompejana*’s thermal limit.

## Materials and Methods

### Animal Collection and Experimentation

**Figure 1:**
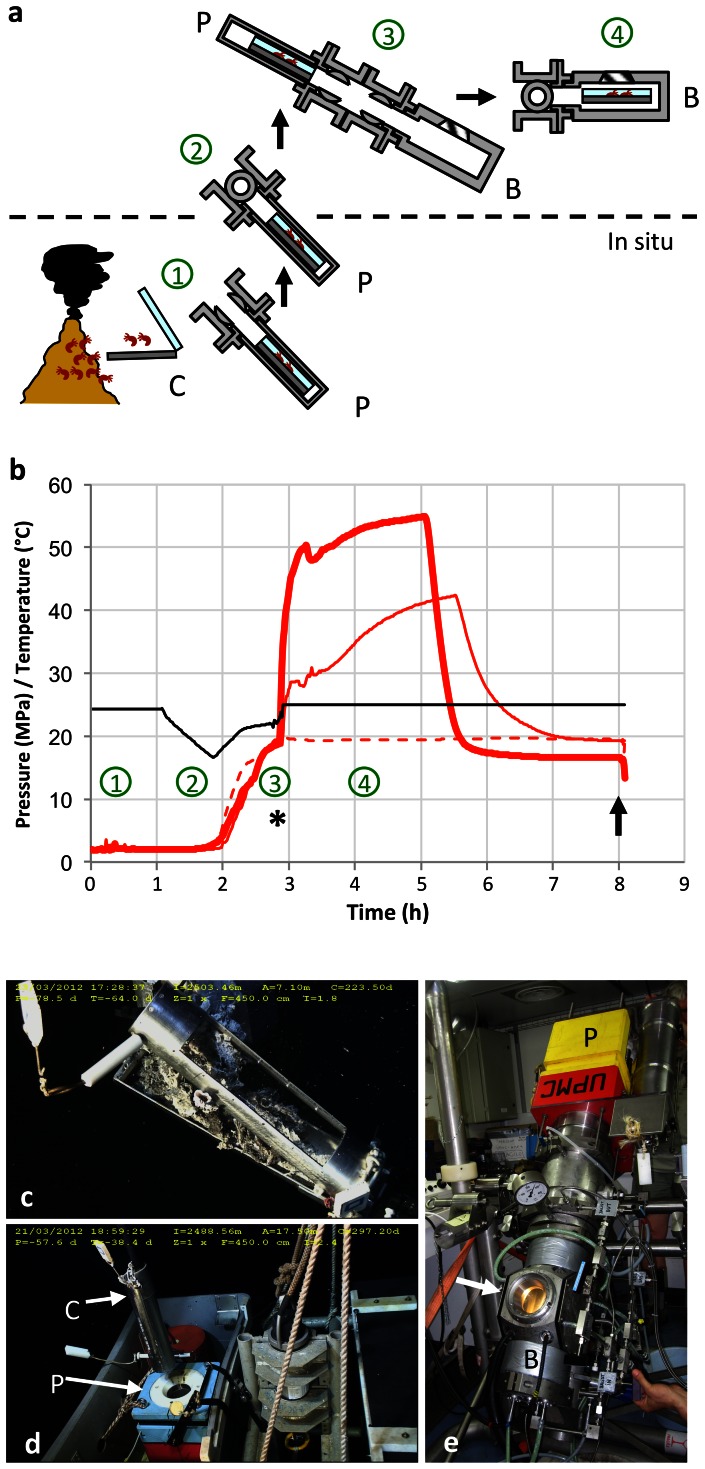
Isobaric sampling and transfer of the Pompeii worms (step = circled number in a and b). **a,** 1: *in situ* sampling of Alvinellas inside a “crocodile” cylinder (C), further closed and inserted in the recovery cell PERISCOP (P). 2: PERISCOP (closed) ascends through the water column. 3: On the ship, samples are transferred from PERISCOP (P) to BALIST (B). 4: thermal exposures. **b,** Pressure (black line) and temperature (red lines) recordings inside the "crocodile" (arbitrary time units). The asterisk and arrow indicate the beginning and end of the 3 thermal exposures (20, 42 and 55°C, dotted, plain and bold lines respectively). Numbers circled in green refer to the steps described in fig. 1a. **c, d,** see a, step 1 (Photographic credit Ifremer/MESCAL 2012). **e**, see a, step 3. A viewport (arrow) allows sample observation inside BALIST.

*Alvinella pompejana* were collected using the DSV *Nautile* (Bio9 and P vent sites, East Pacific Rise, 9°50′N, 2,500 m depth, Mescal 2012 cruise). During 4 different dives, 70 worms were recovered under pressure by using the PERISCOP system [19], composed of an *in situ* sampling cell and an isobaric recovery device. Pompeii worm colonies were sampled as such, and placed inside the sampling cell, using the submersible’s hydraulic arm (Figure 1c). Two autonomous recorders placed inside the sampling cell in direct contact with the samples, provided temperature history during sampling (NKE instruments). Another autonomous recorder provided pressure history, and was checked upon recovery on board of the ship, prior to transfer towards the BALIST aquarium (Text S1, Figure S1). Reproducible pressure profiles remained within 68% (mean value, s.d. = 0.5%, n = 3) to 91% (mean value, s.d. = 2.4%, n = 3) of *in situ* pressure throughout 3 of the recovery processes, and pressure was set to 25 MPa after successful transfer in the BALIST aquarium, in order to proceed to *in vivo* experimental heat-exposures (Figure 1b). In one case however, the pressure profile in PERISCOP was different (reaching a minimum of 52% of *in situ* value during the ascent, before increasing again to 63% upon reaching the surface), and the samples (referred to as ‘sampling’) were processed directly after recovery. Survival of all animals was ascertained through observations of movements, just before sampling their coelomic fluid. The trypan blue exclusion test was employed to determine the percentage of viable coelomocytes (free circulating cells) per individual [20]. Cell counting was performed on board using a Malassez chamber. The animals were further dissected and stored in liquid nitrogen pending analyses. Although not subjected to specific property regulations (international water areas), authors have obtained permission to use samples for any analysis from both chief-scientists. This study did not involve endangered or protected species.

### Microscopy

A portion of the dorsal and ventral regions were fixed in 2.5% glutaraldehyde–seawater solution and post-fixed in 1% osmium tetroxide. Samples were embedded in epoxy resin, semi-thin sections were stained with toluidine blue and observed with a BX61 microscope (Olympus).

### RNA Extraction and Real-time Quantitative RT-PCR (qPCR)

Total RNA was extracted from grounded tissues [21] and controlled for their quality with the Experion® automated electrophoresis system (BioRad) and quantified by spectrophotometry. qPCR analyses were performed as previously described [22] with specific primers designed for *hsp70* gene (*Hsp70* sequence has been deposited with GenBank database under accession number JX560964; sequenced as previously described [22] and assembled with clone Tera 04634 from Alvinella database [12]) and for reference *RPS26* gene (Alvinella database [12]). The *hsp70* expression was normalized to the *RPS26* expression.

## Results and Discussion

**Figure 2:**
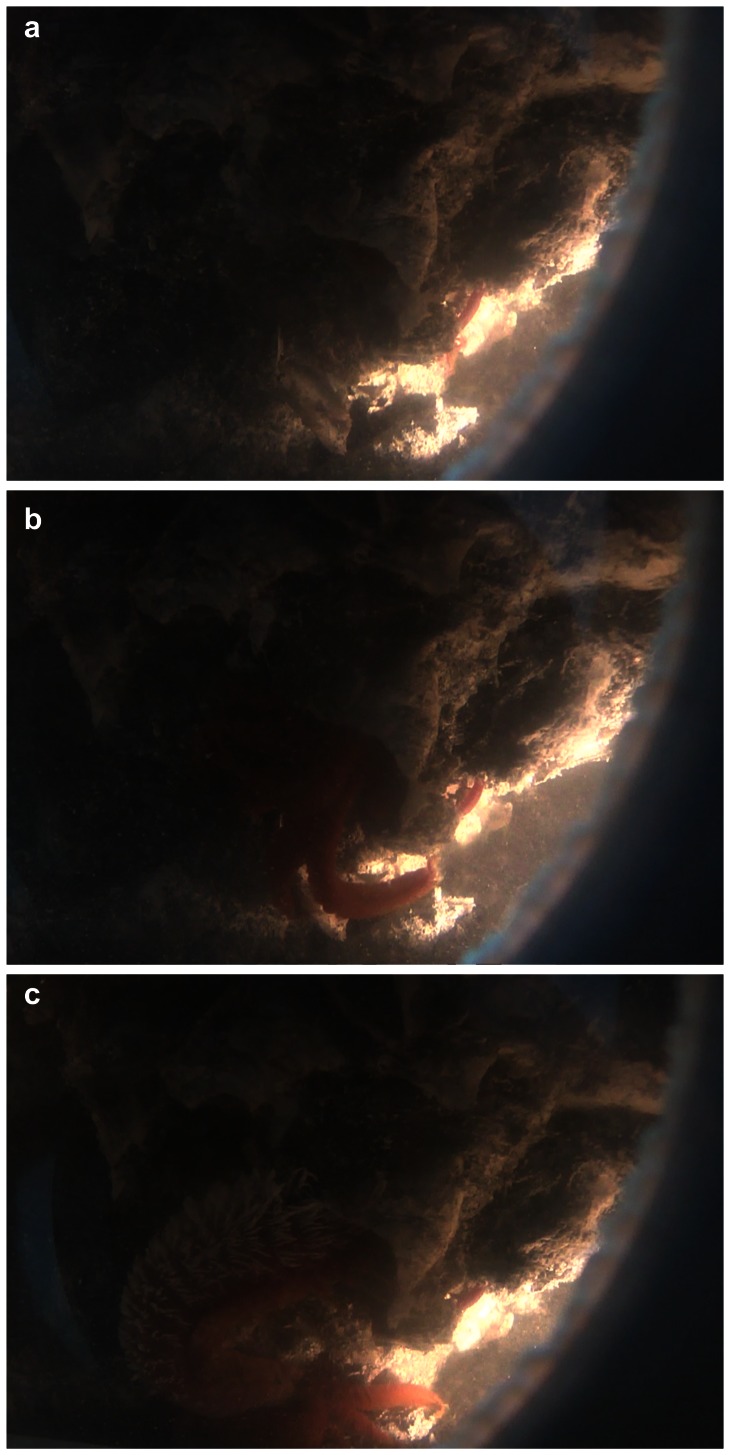
In vivo experiments on *Alvinella pompejana*. Views of a portion of *A. pompejana* tubes inside the BALIST aquarium during the 10 first minutes of the 55°C-exposure experiment. In the middle of the figure, an individual is observed leaving its tube (approximate diameter of tube opening = 1 cm).

**Figure 3:**
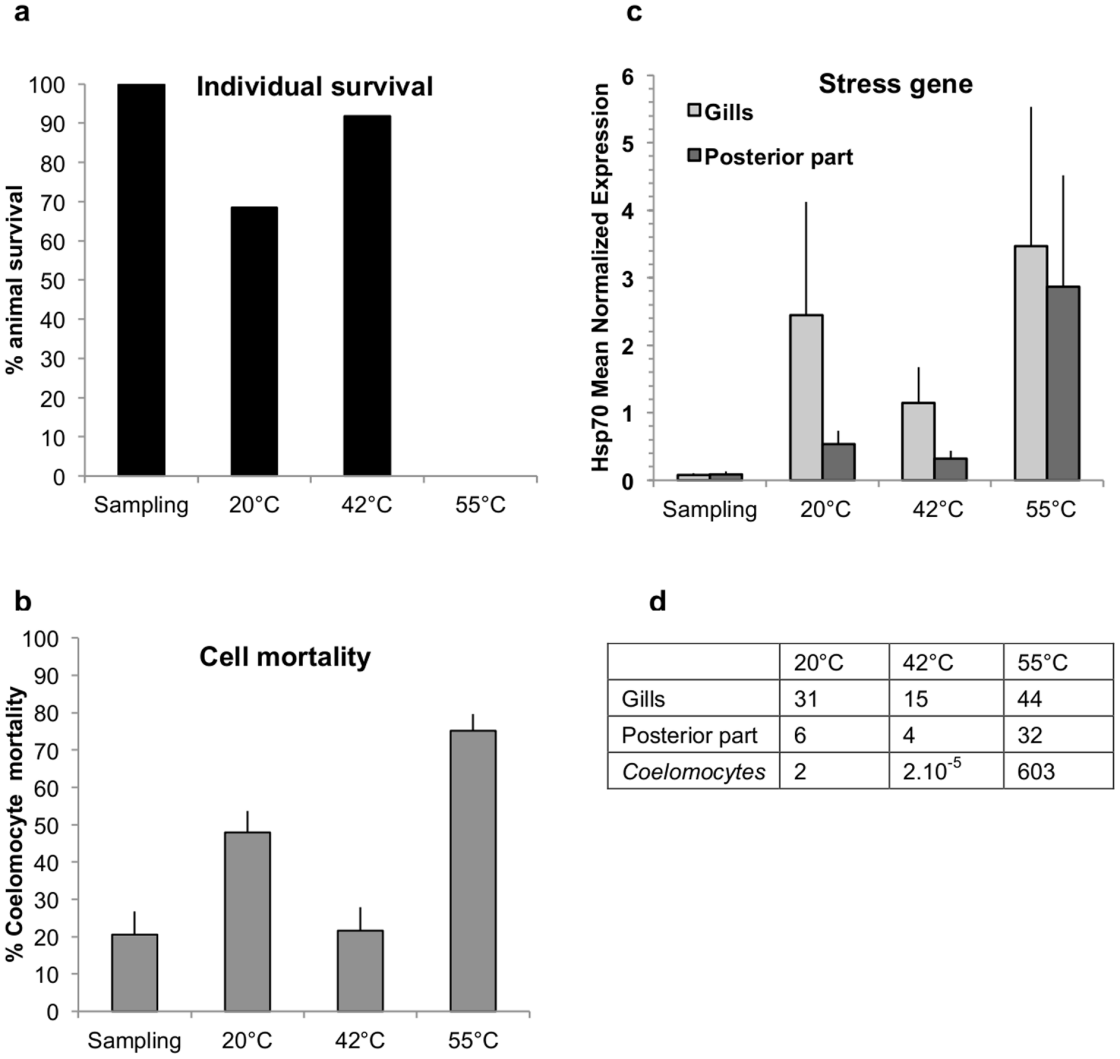
Temperature effect on survival, cell mortality and *hsp70* gene expression in *A. pompejana*. **a**, Survival of *A. pompejana* specimens after the recovery from the PERISCOP sampling device (‘sampling’, n = 9) and the subsequent *in vivo* experiments in the BALIST aquarium (‘20°C’, n = 19; ‘42°C’, n = 24; ‘55°C’, n = 18). **b**, Coelomocytes death in animals from Fig. 3a. **c**, *Hsp70* gene expression (mean for n = 5 individuals ± s.e.m.). Among the four *A. pompejana*’s *hsp70* genes (Alvinella database [12]), two forms showed significant changes according to temperature, and the form that showed the most significant changes is presented here. **d,** Normalized fold *hsp70* expression in gills, posterior part and coelomocytes of experimented animals with respect to ‘sampling’ specimens.

**Figure 4:**
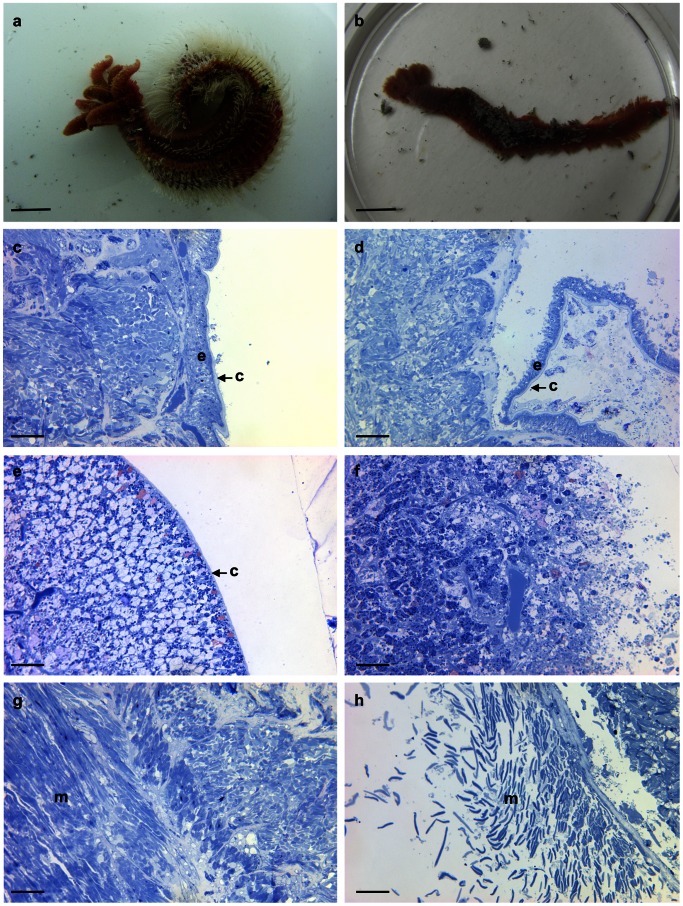
Morphological consequences of a 55°C-exposure in ***A.*** **pompejana** **.**°C-exposed (left column) *versus* 55°C-exposed (right column) specimens. **a, b,** General view of specimens after the *in vivo* experiments; **c, d,** Sections of the dorsal region showing the delamination of the epidermis (e) and cuticle (c) at 55°C; **e, f,** Sections of the ventral tube secreting region showing the disappearance of the tegument and disorganization of the underlying tissues at 55°C; **g, h,** Sections of muscles (m) showing the disorganization of cells at 55°C. Bar represents approximately 1 cm in **a, b** and 50 µm in **c-h**.

Worm colonies collected with the isobaric sampling device and further transferred towards the BALIST high-pressure aquarium, were subjected to three thermal regimes, a constant mild 20°C-exposure, and two heat-exposures followed by a 3 hour-recovery period at 20°C (Figure 1). The heat-exposures lasted about 2 hours, the first one ramped from 30°C to 42°C, and the second one from 50°C to 55°C (thereafter referred to as ‘42°C’ and ‘55°C’ experiments respectively). The behaviour of *A. pompejana* was observed through the aquarium viewport as far as possible, which proved feasible only during the 55°C-exposure because the animals remained inside their tubes during the 20°C-experiment and were not in front of the viewport during the 42°C-experiment. During the first 10 minutes of the 55°C-experiment, individuals of *A. pompejana*, after a short period of ‘normal’ behaviour during which they ventilated their tube by moving up and down, were observed leaving their tubes (Figure 2) and crawling on the surface of the colony. Since *A. pompejana* is extremely sedentary and rarely leaves its tube [3], this unnatural behaviour likely reveals a disturbance. At the end of the experiment at 55°C, all worms (n = 18) were dead (Figure 3a, Figure 4b). They all showed very serious damage of their tissues and cells, with a detachment of the tegument and a disorganization of internal tissues (Figure 4), as well as a high mortality of circulating cells (75±4%, Figure 3b). The mRNA extracted from the tissues (gills and posterior part) and circulating cells of these animals contained a significantly higher quantity of *hsp70* stress gene transcripts in comparison with animals directly processed after sampling (Kruskal-Wallis test, ‘gills’ H = 7.536 p = 0.056, ‘posterior part’ H = 12.40 p = 0.006; post-hoc Mann-Whitney two-sided test for ‘55°C gills’ p = 0.0119 and ‘55°C posterior part’ p = 0.0079; t-test following a mean resampling by bootstrap (n = 100) using the R library ‘stats’, and Bonferroni correction, pairwise t-test p-value<0.0001; Figure 3c and 3d). Since HSP70 proteins are known to be mobilized in response to environmental stresses, among which temperature [23], this confirms that a thermal exposure up to 55°C is harmful for *A. pompejana.* The results of this study provide the first direct empirical evidence that *A. pompejana* cannot withstand prolonged exposure to temperatures in the 50–55°C range, and that its thermal optimum lies below 50°C. Since a higher thermal tolerance was expected from *in situ* measurements, this emphasizes the difficulty of assessing the species thermal tolerances through *in situ* probing in such a stochastic environment.

In contrast with the severe 55°C-heat shock, the 42°C-experiment displayed the highest survival rates at both specimen and cellular levels (92% and 78% respectively; Figure 3a), with no observable structural damage in the tissues (data not shown). The animals did trigger a mild heat stress response with a level of *hsp70* gene expression significantly lower than for the ‘55°C’ specimens (t-test: see previous resampling procedure, pairwise t-test p-value <0.0001; Figure 3c and 3d). Taken together, these data suggest that *A. pompejana* is able to cope with temperatures greater than 40°C, at least for 1 hour, without a high thermal stress response. Regarding the constant 20°C experiment, no obvious structural damage was evidenced (Figure 4). However, specimen survival (13 alive/19 total) was lower than for ‘sampling’ (9/9) or for ‘42°C’ (22/24) individuals (Figure 3a). Besides, cell mortalities were significantly higher in specimens from the 20°C exposure (48%) vs. sampling (20.6%) and 42°C (21.6%) animals (t-test: see previous resampling procedure, pairwise t-test p-value<0.0001; Figure 3b). And finally, stress gene expression followed the same trend with values significantly higher in animals subjected to the ‘20°C’ vs. ‘sampling’ and ‘42°C’ treatments (t-test: see previous resampling procedure, pairwise t-test p-value<0.0001; Figure 3c and 3d). Specimen survival, cell mortality and stress gene expression data therefore show that *A. pompejana* endured more damages subsequent to the 20°C exposure when compared to the 42°C experiment (1h20 above 30°C followed by 1 hour above 40°C). This finding reinforces the idea that *A. pompejana* is a thermophilic species with the lower boundary of its thermal optimum being above 20°C.

With an optimal thermal range expanding from above 20°C to beyond 40°C, *A. pompejana* ranks among the most thermotolerant metazoan species. Interspecies comparisons for thermal limit/tolerance remain however a difficult issue, due to the variety of indexes and protocols used to evaluate the animals’ thermal scope. The highest thermal tolerance limits estimated for any animal were previously reported in the hot springs ostracod *Potamocypris* sp., which survived prolonged exposures to 49°C [24], and in the desert ants *Cataglyphis bombycina* and *Cataglyphis bicolor* based on their critical maximum temperature (CTmax 55°C and 54°C [25]). More recently, the alvinellid *Paralvinella sulfincola* was proven highly thermotolerant based on its preference for temperatures in the 40°–50°C zone and its ability to withstand short exposures at 55°C (thermal limit 50–55°C [17], [18]). *A. pompejana* can be suggested to share a similar thermal preference with the alvinellid *P. sulfincola*, and clearly has a shifted stress response to higher temperatures when compared to the desert ants. The ant response reached its highest point at 37°C and ended around 45°C [25] while *A. pompejana* should yield its highest expression in the 42°C–55°C range. This means that *A. pompejana* only triggers this molecular response upon temperature extremes, and would not have a constantly up-regulated heat shock response, in order to counter-balance the rapid protein denaturation that might have been expected from an animal at the edge of its thermal preference. This was confirmed by the low levels of *hsp70* expression measured in the freshly collected worms (‘sampling’, Figure 3c and 3d) in comparison with the thermal stress response induced during the *in vivo* experiments, and especially the 55°C-experiment. Moreover, the low natural vs. *in vivo* stimulated levels of *hsp70* expression mean that these ‘sampling’ worms had not experienced heat stress during the hours that preceded the collection. A comparable result was previously obtained in another vent chimney dweller, the Atlantic shrimp *Rimicaris exoculata* [21]. As proposed for other vent species, these animals may prefer temperatures well within their tolerated range, thereby maintaining a ‘safety margin’ against rapid temperature fluctuation that could expose them to thermal extremes [26].

The extreme temperature variability in the close surrounding of *A. pompejana*, and notably the sharp thermal gradient the animal is believed to endure in its tube (up to 60°C from the opening to the bottom) [3], [6], have led to propose this animal as one of the most eurythermal on earth. The *hsp70* gene analyses revealed a differential expression in the anterior and posterior parts of the worms (‘20°C’ and ‘42°C’ specimens; Figure 2c), the posterior part responding less to thermal variations than the gills (One-way ANOVA across treatments following bootstrap resampling (n = 100) performed on gills (F = 27.84, p-value = 2 e^−7^) and posterior part (F = 5.42, p-value = 0.02)). Added to previous results on differential cell membrane compositions [27], this tends to confirm the existence of such a thermal gradient inside the tube and consequently the exceptional eurythermy of *A. pompejana*.

In conclusion, while *A. pompejana* is not as thermophilic as previously suspected, it nevertheless remains among the most thermotolerant and eurythermal metazoans. The accurate definition of the metazoan UTL will further require standardized indexes, like the CTmax (i.e. a behavioural response [28]), to be provided for alvinellids. Since these tubicolous animals may retract inside their tubes, or move behind the tube masses, behavioural studies are challenging, and future isobaric sampling processes could aim at collecting Alvinella worms without their tubes. Finally, isobaric sampling and transfer should allow many more deep-sea species to be studied alive. Such studies are urgently needed to better understand the biological responses of deep fauna to environmental changes, in times when evidence for anthropogenic impact on the world's largest ecosystem is accumulating [29].

## Supporting Information

Figure S1 **General schematic view of the BALIST aquarium**. For detailed legend see Text S1.
(TIF)

Text S1 **Description of the BALIST aquarium**.
(DOCX)
