# Supplementary figures and images for: Thermal Limit for Metazoan Life in Question: In Vivo Heat Tolerance of the Pompeii Worm
Source: PLoS One. 2013 May 29;8(5):e64074. doi: 10.1371/journal.pone.0064074 (PMC3667023; doi:10.1371/journal.pone.0064074)

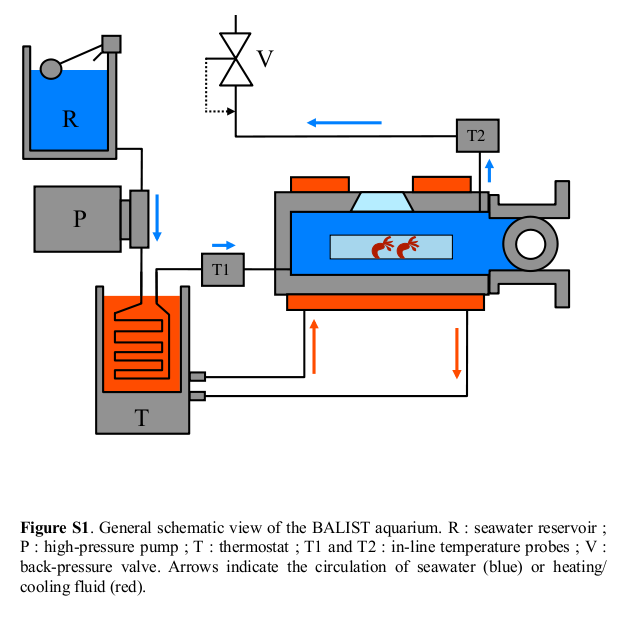

Supplement: Figure S1 — General schematic view of the BALIST aquarium. For detailed legend see Text S1. (TIF) [file pone.0064074.s001.tif]
