# Supplementary material for: Thermal Limit for Metazoan Life in Question: In Vivo Heat Tolerance of the Pompeii Worm
Source: PLoS One. 2013 May 29;8(5):e64074. doi: 10.1371/journal.pone.0064074 (PMC3667023; doi:10.1371/journal.pone.0064074)
Supplement: Text S1 — Description of the BALIST aquarium. (DOCX) [file pone.0064074.s002.docx]

**Text S1**. **Description of the BALIST system**.

The BALIST pressurized device consists in a pressurised vessel of approximately 8 L internal volume, composed of X4CrNiMo16-5-1 stainless steel. As shown in Figure 1e, BALIST provides a large 8 cm-diameter sapphire viewport for direct observation, and smaller viewports for providing illumination. It functions at pressures up to 300 bars (equivalent to approx. 3000 m depths), and at temperatures ranging from 2 to 100°C. BALIST (Figure S1) is a flow-through system which circulates filtered seawater (1 µm mesh), with flow rates that may reach 10 L / hour. A 60 L seawater reservoir feeds a high-pressure pump (Axflow, Plaisir, France), which injects pressurised water in the BALIST. Beyond the latter, further down the line, the pressure is controlled by manually operating a back-pressure valve (TESCOM LAA, Aix, France). The oxygen content of the seawater is kept low, as in deep-sea waters surrounding vents (Shillito et al 2001), by bubbling a 95.5 / 4.5% N_2_ / O_2_ gas mixture in the supply reservoir. The temperature of the seawater in BALIST is controlled by a thermostat, which allows the circulation of a cooling/heating fluid around the experimental vessel, and around the feeding line, prior to seawater entry inside BALIST. The temperature of circulating seawater is measured in the in- and out-going water lines, and is displayed on digital readers. These readings are used as proxys to evaluate the temperature prevailing inside the "crocodile" sampling cell. The exact experimental temperature, mentioned throughout the manuscript, is later obtained using the mean value of the two autonomous temperature loggers, which are inside the sampling cell.

One specific feature of BALIST is that it is equipped with a ball valve providing a 10 cm diameter passage, identical to that of PERISCOP (Shillito et al 2008), allowing isobaric coupling of the 2 devices. Prior to isobaric transfer, both devices are coupled by means of 3 collar-shaped clamps, which fasten around shoulderings of their respective ball-valve casings. Pressure may further be equilibrated between PERISCOP, BALIST, and the volume enclosed between the 2 devices, according to pressure measured inside PERISCOP when it is recovered. Once this is achieved, the ball valves may be manually operated, thereby allowing transfer of the "crocodile" sampling cell, and final adjustment of pressure to experimental conditions (25 MPa). As shown in Figure 1e, BALIST is positioned on a articulated stainless-steel frame, allowing the coupled devices to be manually tilted, in order to transfer the "crocodile" by gravity. Once the transfer is achieved, the frame can be returned and locked in the horizontal position.

Shillito B, Jollivet D, Sarradin PM, Rodier P, Lallier F, et al. (2001) Temperature Resistance of *Hesiolyra bergi*, a Polychaetous Annelid Living on Vent Smoker Walls. Mar Ecol Prog Ser 216: 141-149.

Shillito B, Hamel G, Duchi C, Cottin D, Sarrazin J, et al. (2008) Live capture of megafauna from 2300 m depth, using a newly designed Pressurized Recovery Device. Deep-Sea Res I 55: 881-889.
